# Evaluation of children with macrocytosis: clinical study

**DOI:** 10.11604/pamj.2018.31.54.15498

**Published:** 2018-09-25

**Authors:** Hakan Sarbay, Yılmaz Ay

**Affiliations:** 1 Diyarbakır Children Hospital, Paediatric Hematology and Oncology Diyarbakır, Turkey; 2 Pamukkale University Faculty of Medicine, Paediatric Hematology and Oncology, Turkey

**Keywords:** Macrocytosis, bone marrow failure, acute leukemias

## Abstract

**Introduction:**

in this study, it is aimed to emphasize the causes and importance of macrocytosis in paediatric practice.

**Methods:**

in the paediatric hematology and oncology clinic, 1752 patients evaluated in 2017, patients with macrocytosis were examined retrospectively with clinical and laboratory findings. Patients with macrocytosis were compared with the frequency of severe hematologic diseases such as acute leukemia and bone marrow failure in patients with normocytic and microcytic mean corpusculer volume.

**Results:**

macrocytosis was detected in 72 out of 1752 patients (4.1%) in the study. Acute lymphoblastic leukemia in 11(15.2%), acute myeloid leukemia in 3(4.1%), Fanconi aplastic anemia in 7(9.7%), Diamond-Blackfan anemia in 2(2.7%), aplastic anemia in 1(1.3%), congenital diseritropoetic anemia in 1(1.3%), deficiency of vitamin B12 in 14(19.4%) were diagnosed. Down syndrome was presented in 33 cases (45.8%). The number of patients with acute leukemia diagnosis was 14(0.8%) in the group in which the mean corpusculer volume was either microcytic or normocytic. The incidence of acute leukemia and bone marrow failure was significantly higher in the group with macrocytosis compared between the two groups.

**Conclusion:**

although vitamin B12 deficiency is considered firstly in patients who have undergone cytopenia and macrocytosis in our country, haematological malignancies, bone marrow failures, myelodysplasia and myeloproliferative diseases should be remembered especially in the individuals with Down syndrome at the same time.

## Introduction

Erythrocytes are classified into three morphological groups by their mean cell volume (MCV): microcytic, normocytic, and macrocytic. Macrocytosis is the enlargement of erythrocytes more than 2 standard deviations than the normal value of the age group. The classification by MCV also constitutes the foundation of anemia classification used mostly in clinical practice. Although MCV values vary by age and sex, reticulocyte count and other series are considered important for the examination of patients [1]. Macrocytic anemias are classified into two groups: one is associated with the megaloblastic changes in bone marrow and the other is non-megaloblastic anemia characterized by macrocytosis. Ninety five percent of megaloblastic anemias are caused by deficiency of vitamin B12 and/or folate. Whereas the most frequent causes of non-megaloblastic macrocytosis are neonatal period, reticulocytosis, liver dysfunction, aplastic anemias, and myelodysplastic syndromes [2, 3]. The aim of the present study is to underline the causes and significance of macrocytosis in clinical paediatric practice in Pamukkale University Faculty of Medicine and Diyarbakir Children Hospital Paediatric Hematology-Oncology clinics.

## Methods

Retrospective analysis of the clinical and laboratory findings of 1752 patients, who were examined in the paediatric hematology and oncology outpatient clinics in a one year period during January-December 2017 were included. To identify the etiology, blood count, peripheral smear, bone-marrow aspiration and biopsies, erythrocyte sedimentation rate, kidney-liver-thyroid function tests, a-fetoprotein (AFP), hemoglobin F (HbF), vitamin B12 and folate levels were evaluated. Patients whose MCV values were more than 2 standard deviations than the normal value of the age group were grouped in macrocytosis. Etiology was investigated in these patients. There was any exclusion criteria in the study and variable datas are collected from our clinic archive. Prevalence rates of any severe hematological diseases such as acute leukemia and bone marrow deficiency were compared between macrocytic and normocytic or microcytic groups. Ethics committee approval was obtained for the study (30.01.18/03).

**Statistical analyses:** all the descriptive analyses were performed by Statistical Package for the Social Sciences 22.0 (SPSS Inc.; Chicago, IL, USA). For the comparison of the categorical variables, Fisher Chi-Square test was used.

## Results

Macrocytosis was detected in 72 of 1752 patients. The mean age of patients with macrocytosis were 5.7 (3 months to 17 years) years and 44(61.2%) were male and 28(38.2%) were female. Eleven patients (15.2%) were diagnosed with acute lymphoblastic leukemia (ALL), 3(4.1%) with acute myeloid leukemia (AML), 7(9.7%) with Fanconi aplastic anemia (FAA), 2(2.7%) with Diamond-Black fan anemia (DBA), one (1.3%) with aplastic anemia, one (1.3%) with congenital dysery thropoietic anemia (CDA), and 14(19.4%) with vitamin B12 deficiency. In 33(45.8%) of the cases, the diagnosis of Down syndrome (DS) was present. Patients with ALL consisted of 3 T-cell ALL and 8 pre-B cell ALL. Types of patients with AML were M0, M6 and M7. The most frequent cause for presentation and referral is the suspected cytopenia and malignancy. No history of a chronic disease except DS and no established drug use in the etiology of macrocytosis were present. Vitamin B12 and folate deficiency diagnosis was based on serum levels of vitamin B12 (normal value >200 pg/ml), folate (normal value>4.1 ng/ml), hemogram, and the findings of peripheral smear (hypersegmented neutrophils-the presence of neutrophils with six or more lobes or the presence of more than 3% of neutrophils with at least five lobes) and clinical evaluation. When 10 patients with bone marrow deficiency were evaluated, the high levels of HbF were detected in all, only mild high levels of AFP was found in only 2 patients with FAA.

In the clinical and laboratory investigations of three (4.1) cases, no underlying cause that may lead to macrocytosis and no associated cytopenia were established. In 28 (84.8%) of 33 patients with DS and two (14.2%) of 14 patients with vitamin B12 deficiency no signs for cytopenia were observed. Of 25 patients with acute leukemia and bone marrow deficiency one had isolated mild thrombocytopenia, three had anemia, one neutropenia-leukopenia, and the remaining 20(80%) had either bicytopenia or pancytopenia. While one patient with acute leukemia had a moderate increase in liver function tests, one patient with DS was having treatment for hypothyroidism (Table 1). The number of patients diagnosed with acute leukemia was 14(0.8%) in microcytic or normocytic group (n=1680) by MCV values. Although patients with pancytopenia or bicytopenia caused by infections were included, they were not given the diagnosis of bone marrow deficiency. In the comparison of the macrocytic group to microcytic or normocytic group by MCV values, the prevalence of acute leukemia and bone marrow deficiency was found significantly higher in macrocytosis group (p< 0.001) (Table 2). In the evaluation of the patients with Down syndrome within itself, of 106 patients with DS, 33(33.1%) had macrocytosis. Two patients with macrocytosis were diagnosed with AML-M6 and ALL at the time of the first admission. One patient was diagnosed with myelofibrosis based on bone marrow biopsy examined upon cytopenia and hypocellularity of bone marrow aspiration confirmed at outpatient follow up visit. This patient developed AML-M7, identified at follow up after 7 months. The comparison between the groups with and without macrocytosis showed that the prevalence of acute leukemia was significantly higher in patients with DS (Table 3).

**Table 1:** characteristics of patients with macrocytosis, acute leukemia and bone marrow failure

Patient	Diagnosis	Age	Gender	Wbc /mm^3^	Neut /mm^3^	Hgb gr/dl	MCV	Plt /mm^3^	Physical examination	LFT	TFT	B12-folat
1	AML M0	8	Male	41.990	8100	7,1	99	24.000	HSM	N	-	N
2	AML M6-DS	2	Female	12.980	1200	5,6	93	50.000	HSM	N	N	N
3	ALL	11	Female	44.780	440	5,4	94	14.000	HSM-LAP	N	-	N
4	ALL	4	Male	8270	180	7,5	89	24.000	HSM-LAP	N	N	N
5	ALL	15	Male	12.290	290	3,7	94	6000	HSM-LAP	N	N	N
6	ALL	8	Female	7670	1160	7,3	93	263.000	HSM-LAP	N	N	N
7	ALL	4	Male	7470	210	7,2	101	171.000	HSM-LAP	N	-	-
8	ALL	6	Female	12.690	260	7,3	90	26.000	HSM-LAP	N	-	-
9	ALL	10	Male	3920	270	6,6	94	67.000	HSM	N	N	-
10	ALL	3	Female	3320	600	11,4	89	193.000	HSM	2 fold increase	N	N
11	ALL	7	Male	39.630	540	6,6	94	34.000	HSM-LAP	N	-	N
12	ALL	12	Female	57.980	1370	3,4	107	67.000	HSM-LAP	N	N	N
13	ALL-DS	6	Female	26.850	880	5,5	102	25.000	HSM-LAP	N	N	N
14	FAA	11	Female	1860	220	8,5	98	38.000	GR, Cafe-au-lait spots	N	N	N
15	FAA	5	Male	3810	330	8,2	94	26.000	GR, Cafe-au-lait spots	N	N	N
16	FAA	4	Male	5710	1180	12	96	144.000	GR	N	N	N
17	FAA	5	Male	4020	2030	12,2	93	74.000	GR, Cafe-au-lait spots	N	N	N
18	FAA	5 Month	Male	9820	3560	5,4	101	323.000	microcephaly	N	N	N
19	FAA	6	Female	4320	1840	11,7	96	26.000	GR, Cafe-au-lait spots	N	N	N
20	FAA	2	Female	3330	1340	10,2	90	65.000	GR, Cafe-au-lait spots	N	N	N
21	AA	14	Female	2190	470	8,2	100	32.000	-	N	N	N
22	DBA	2	Male	5260	1520	7,5	103	462.000	GR	N	N	N
23	DBA	1,5	Female	9920	2820	6	89	446.000	-	N	N	N
24	CDA	13	Female	4740	3470	2,5	104	240.000	-	N	N	B12: 146
25	MFS-AML M7-DS	11 Month	Male	3170	890	5	94	17.000	HSM	N	N Treated for hypothyroidism	N

Wbc: White blood cell count, neut: neutrophil count, hgb: hemoglobin, plt: thrombocyte count, MCV: Mean corpusculer volume, LFT: liver function tests, TFT:Thyroid function tests, AML: acute myeloid leukemia, ALL: acute lymphoid leukemia, FAA: Fanconi aplastic anemia, AA: aplastic anemia, DBA: Diamond–Blackfan anemia, CDA: congenital dysery thropoetic anemia, MFS: myelofibrozis, DS: Down syndrome, HSM: hepatosplenomegaly, LAP: lymphadenopathy, GR: Growth retardation

**Table 2:** comparison of the incidence of acute leukemia and bone marrow failure

	Patients diagnosed with acute leukemia and bone marrow failure	Patients without acute leukemia and bone marrow failure	Total case	pvalue
Macrocytosis	25	47	72	p<0,001
Microcytic, normocytic	14	1666	1680	

Chi-square test

**Table 3:** comparison of the incidence of acute leukemia and myelodysplastic syndrome between patients with macrocytosis and without macrocytosis in Down syndrome

	Patients diagnosed with acute leukemia and MDS	Patients without acute leukemia and MDS	Total case	pvalue
Macrocytosis	3	30	33	0,028
Microcytic, normocytic	0	73	73	

Fisher Ex: test

## Discussion

Macrocytosis is caused by four main mechanisms. These include nutritional deficiencies, bone marrow disorders, medication, and chronic diseases [4]. Macrocytosis is rarely observed in children compared to normocytic or microcytic conditions, based on MCV [5]. In our study, the prevalence of macrocytosis was 4.1% in 1752 patients. Although there are no studies on frequency in children in the literature, macrocytosis prevalence estimates ranging from 1.7% to 3.6% in adults [5, 6]. Our study had almost similar results when compared to the literature. Macrocytic anemias are classified as megaloblastic and non-megaloblastic. Non-megaloblastic anemia is associated with liver disease, hypothyroidism, bone marrow deficiencies, and MDS. The most common cause of megaloblastic anemia in children is vitamin B12 deficiency [5]. In our study, patients diagnosed with vitamin B12 deficiency were either suffering from accompanying iron deficiency or carriers of thalassemia, macrocytosis could only be identified in 14 patients. Failing to assess the utilization tests for vitamin B12 such as the measurements of serum concentrations of homocysteine and methylmalonic acid is considered as a limitation in diagnosing vitamin B12 deficiency. Pappo et *al*. [7] in their study in 146 children identified the causes of macrocytosis as medication (51 patients), congenital heart disease (20), Down syndrome (12), bone marrow deficiency or MDS (6), and various chronic diseases (21). However, in 24 of them the cause of anemia could not be determined. In our study, acute leukemia, bone marrow deficiency, Down syndrome, and vitamin B12 deficiency were the leading causes. No history of medication was present. The reason the distribution of diagnoses differed in our study than that of Pappo et *al*. study has been considered as the result of overlooking of MCV values in patients without cytopenia and failing to refer them to paediatric hematology outpatient clinics. Erythrocytes in neonatal period tend to be physiologically macrocytic. In post neonatal period the upper limit of MCV decreases to 85 fL; over 6 months of age, the upper limit is calculated with MCV=84+0.6xAGE formula [8]. Patients in neonatal period were not included in our study. Individuals with Down syndrome also constitute a patient group with high prevalence for macrocytosis. In evaluation studies, macrocytosis was the most frequently obtained hematological finding in DS [9]. In a study by Prasher et *al*., macrocytosis was identified as the most frequent symptom in 147 adult patients [10]. In our cases, 33 of 106 patients with DS were determined to have macrocytosis. Two patients with macrocytosis had lower levels of vitamin B12. Since the levels of vitamin B12 and folic acid measurements were not performed in all DS cases with macrocytosis, no correlation between vitamin B12 and folic acid in this group of patients could be established. However, in previous studies, vitamin B12, and folic acid deficiency, and increased levels of hemoglobin F have not been determined as the underlying cause of macrocytosis in individuals with DS [11].

Macrocytosis may be encountered as the earliest symptom in bone marrow deficiencies, and in MDS. Macrocytosis may be detected even in cases with normal blood count, and responsive to treatment [12]. Macrocytosis and thrombocytopenia FAA are the most frequently obtained laboratory findings. Cytopenias usually occur at around the age of 5-10 years with a mean age of 7. In patients presenting with a congenital anomaly, and growth and developmental delay, if macrocytosis is present in blood count, then FAA should be suspected [13]. DBA and CDA are bone marrow deficiency syndromes, of which the former is associated with erythroid cell aplasia, and the latter is characterized by morphological abnormalities in erythrocytes and by ineffective erythropoiesis that affects isolated erythroid series. Majority of the patients have macrocytic anemia [14]. In our study, seven FAA, two DBA, one aplastic anemia, and one CDA patient had cytopenia and macrocytosis in their blood counts performed for diagnostic assessment (Table 1). Acute leukemia is the most common malignity of childhood accounts for 1/3 of all childhood malignancies. Although the etiology of acute leukemia has not been understood clearly, genetical abnormalities mainly include aberrations in karyotypes, chromosomal translocations, and deletions. Typical examples are Down syndrome and FAA. Laboratory findings may include leukocytosis, leukopenia, thrombocytopenia, and anemia. Anemia usually takes the form of normochromic normocytic anemia accompanied with reticulocytopenia [14]. In our study, of 28 patients diagnosed with acute leukemia, 14 (50%) had macrocytosis, and 14 (50%) had microcytic or normocytic MCV values. The prevalence for acute leukemia was higher in patients with macrocytosis (Table 2). Compared to normal population, children with DS have an increased risk of developing acute leukemia and MDS [15]. Majority of the patients with lymphoid leukemia show immuno-phenotypic features of precursor B cell ALL. In patients with DS, unlike the myeloid leukemia, lymphoid leukemias are not observed during the period of infancy. Due to intrinsic drug resistance and treatment related mortalities, prognosis was worse than that of lymphoid leukemia, which occurs sporadically [16, 17]. The incidence of AML was found to be 150 times more in children with DS. The most common form of AML in children with DS is the AML-M7 acute mega karyoblastic leukemia [18]. AML-M6 and AML-M1/M2 are less frequent than AML-M7 in patients with DS [19, 20]. In our 106 cases with DS, three of them developed acute leukemia. They were diagnosed with AML-M6, AML-M7 and ALL. Within the patients with DS, comparison between the groups with and without macrocytosis showed a significantly higher incidence of acute leukemia (Table 3).

## Conclusion

Macrocytosis is a significant symptom in the diagnosis of bone marrow diseases and acute leukemia in childhood. Although, given the present conditions of our country, vitamin B12 deficiency is the first suspect in patients with cytopenia and macrocytosis, yet haematological malignancies, bone marrow deficiencies (congenital or acquired), myelodysplasia, and myeloproliferative diseases should be borne in mind especially in the individuals with Down syndrome, as well.

### What is known about this topic

- Macrocytosis is rarely observed in children compared to normocytic or microcytic conditions, based on MCV;
- The most common causes in the literature are drugs and chronic diseases;
- The number of studies done on children about macrocytosis and etiology is not enough.

### What this study adds

- Macrocytosis can be an alarm finding for suspicion of bone marrow pathologies;
- In individuals with Down syndrome, patients with macrocytosis should be more closely monitored for myelodysplastic syndrome and acute leukemia.

## Competing interests

The authors declare no competing interests.

## Authors’ contributions

All authors participated in the design of the study. Hakan Sarbay performed the collection, statistical analysis and interpretation of the data. Yılmaz Ay participated to the interpretation of data. All authors have read and approved the final document.
